# Adiposity influences on myocardial deformation: a cardiovascular magnetic resonance feature tracking study in people with overweight to obesity without established cardiovascular disease

**DOI:** 10.1007/s10554-023-03034-2

**Published:** 2024-02-02

**Authors:** Constantin Bolz, Edyta Blaszczyk, Thomas Mayr, Carolin Lim, Sven Haufe, Jens Jordan, Philipp Barckow, Jan Gröschel, Jeanette Schulz-Menger

**Affiliations:** 1 Charité Universitätsmedizin Berlin, Working Group on Cardiovascular Magnetic Resonance, Experimental and Clinical Research Center, A Joint Cooperation Between the Charité Universitätsmedizin Berlin and the Max-Delbrück-Center for Molecular Medicine, Berlin, Germany; 2 DZHK (German Centre for Cardiovascular Research), Partner site Berlin, Berlin, Germany; 3 Helios Hospital Berlin-Buch, Department of Cardiology and Nephrology, Berlin, Germany; 4 Clinic for Rehabilitation and Sports Medicine, Hannover Medical School, Hannover, Germany; 5 Institute of Aerospace Medicine, German Aerospace Center, Cologne, Germany; 6 Circle Cardiovascular Imaging Inc., Calgary, Alberta Canada

**Keywords:** Obesity, Weight loss, Myocardial deformation, Feature tracking, Left atrial enlargement, Diastolic dysfunction

## Abstract

The objective of this study was to assess whether dietary-induced weight loss improves myocardial deformation in people with overweight to obesity without established cardiovascular disease applying cardiovascular magnetic resonance (CMR) with feature tracking (FT) based strain analysis. Ninety people with overweight to obesity without established cardiovascular disease (age 44.6 ± 9.3 years, body mass index (BMI) 32.6 ± 4 kg/m^2^) underwent CMR. We retrospectively quantified FT based strain and LA size and function at baseline and after a 6-month hypocaloric diet, with either low-carbohydrate or low-fat intake. The study cohort was compared to thirty-four healthy normal-weight controls (age 40.8 ± 16.0 years, BMI 22.5 ± 1.4 kg/m^2^). At baseline, the study cohort with overweight to obesity without established cardiovascular disease displayed significantly increased global circumferential strain (GCS), global radial strain (GRS) and LA size (all *p* < 0.0001 versus controls) but normal global longitudinal strain (GLS) and normal LA ejection fraction (all *p* > 0.05 versus controls). Dietary-induced weight loss led to a significant reduction in GCS, GRS and LA size irrespective of macronutrient composition (all *p* < 0.01). In a population with overweight to obesity without established cardiovascular disease subclinical myocardial changes can be detected applying CMR. After dietary-induced weight loss improvement of myocardial deformation could be shown. A potential clinical impact needs further studies.

**Supplementary Information:**

The online version contains supplementary material available at 10.1007/s10554-023-03034-2.

## Introduction

Obesity is a major public health issue with nearly a third of the world population being classified as a person with overweight or obesity [1, 2]. Obesity increases the risk for hypertension and for coronary artery disease (CAD), which have a strong association with the development of heart failure (HF) [3]. Furthermore, obesity independently predicts HF [4]. In particular, obesity predisposes to HF with preserved ejection fraction (HFpEF) [5–8]. Obesity-associated volume overload, neurohumoral activation, relative natriuretic peptide deficiency, myocardial lipotoxicity, and systemic proinflammation could conceivably contribute to myocardial remodeling and diastolic dysfunction [3, 9]. Assessing asymptomatic diastolic dysfunction in people with overweight to obesity without established cardiovascular disease is important to identify risks of progressing to overt HF early on and to individualize preventive measures [10–12].

Echocardiographic parameters are commonly assessed to evaluate diastolic dysfunction and are recommended by several HF guidelines [13, 14]. The role of cardiovascular magnetic resonance (CMR) in assessing diastolic function is still evolving, despite its well-known versatility and wide range of quantitative parameters in cardiovascular medicine [15, 16]. Recent studies suggested that myocardial deformation using different CMR techniques as well as left atrial (LA) size could identify diastolic dysfunction in HFpEF patients [17–19]. CMR with feature tracking (FT) analysis enables the quantification of myocardial deformation by strain analysis based on routinely acquired steady-state free precession (SSFP) CMR images [20]. CMR-FT has been applied in different cardiovascular diseases suggesting that it has incremental prognostic value for major adverse cardiovascular events and all-cause death in acute myocardial infarction [21], ischemic and nonischemic dilated cardiomyopathy [22], diabetes [23] and HF [24] surpassing widely used cardiac parameters like LV ejection fraction. It has been also linked to reverse cardiac remodeling in patients with severe aortic stenosis following transcatheter aortic valve replacement [25]. Some studies showed the potential of CMR-FT to detect subclinical myocardial dysfunction in different pathologies [26–29]. Other CMR techniques to assess myocardial deformation are myocardial tagging, strain-encoded imaging (SENC) and displacement encoding with stimulated echoes (DENSE) [30–32]. Some echocardiographic studies analyzing cardiac remodeling in people with obesity without overt cardiovascular disease undergoing dietary or bariatric surgical intervention showed an improvement in diastolic function through intentional weight loss [33–35]. Similarly, CMR studies in healthy people with obesity who intentionally lost weight found concomitant reductions in left ventricular (LV) mass [36–38], and LA volume [39]. However, reports regarding the effect of dietary intervention on CMR-derived LV strain are scarce in people with overweight to obesity without established cardiovascular disease [34, 39].

Therefore, we applied CMR to determine whether dietary-induced weight loss improves myocardial deformation in people with overweight to obesity without established cardiovascular disease.

## Materials and methods

### Study population

The study population originates from the B-SMART study which was conducted between March 2007 and June 2010 (Berlin Study of Metabolomics in Adiposity and its Role for Successful Therapy) (ClinicalTrials.gov Identifier: NCT00956566). The B-SMART protocol has previously been described in detail [40]. Briefly, adults with a body mass index (BMI) ≥ 27 kg/m^2^, a sedentary lifestyle (physical activity less than 2 h per week), and no regular medication were included. Exclusion criteria were type 2 diabetes, history of CV disease, hypertension, pregnant or nursing women, and standard contraindications to magnetic resonance. Subjects were randomly assigned to one of two hypocaloric diets for 6 months: Low-carbohydrate or low-fat diet. In both dietary groups total energy intake was reduced by 30% of the baseline food protocol to a minimum of 1,200 kcal per day. Cardiovascular assessments were performed at baseline and after 6-month dietary intervention. The study was approved by our institutional ethical board of Charité and informed written consent was obtained from each subject. We compared the study population with overweight to obesity without established cardiovascular disease to healthy normal-weight controls (BMI 18.5–24.9 kg/m^2^) who have been described elsewhere [41].

### CMR protocol

The study was performed on a 1.5 Tesla MR scanner (Sonata and Avanto, Siemens Medical Solutions AG, Erlangen, Germany). After initial anatomic scout images had been obtained, we performed high temporal resolution cine imaging with a balanced steady-state free precession sequence (repetition time = 16.3 ms, echo time = 1.15 ms, 64 phases, matrix 208 × 256, field of view 325 × 400 mm^2^, in plane resolution 1.6 × 1.6 mm^2^, retrospective ECG-gating). During repetitive breath-holds in end-expiration we acquired a stack of contiguous short-axis slices from the atrioventricular ring to the apex (slice thickness 7 mm, interslice gap﻿ 3 mm) and 2-chamber, 3-chamber and 4-chamber views in long-axis.

### CMR postprocessing analysis

Using commercially available postprocessing software (CVI^42^, version 4.1.2, Circle Cardiovascular Imaging Inc., Calgary, Canada) LV function, volumes and mass were retrospectively quantified in a whole short-axis stack according to the recommendation of the Society for Cardiovascular Magnetic Resonance (SCMR) [16]. These LV parameters have already been published [37], but assessment of FT based strain analysis was performed now. Furthermore, LA quantification was performed, based on the biplanar approach using 2-chamber and 4-chamber view as recently published [42]. Images were analyzed in LA diastole and systole to assess LA size and function. All these measures were indexed to height. The comprehensive acquisition protocol, sequence parameters and postprocessing analysis of the control group have already been reported [41].

### Feature tracking analysis

**Fig. 1:**
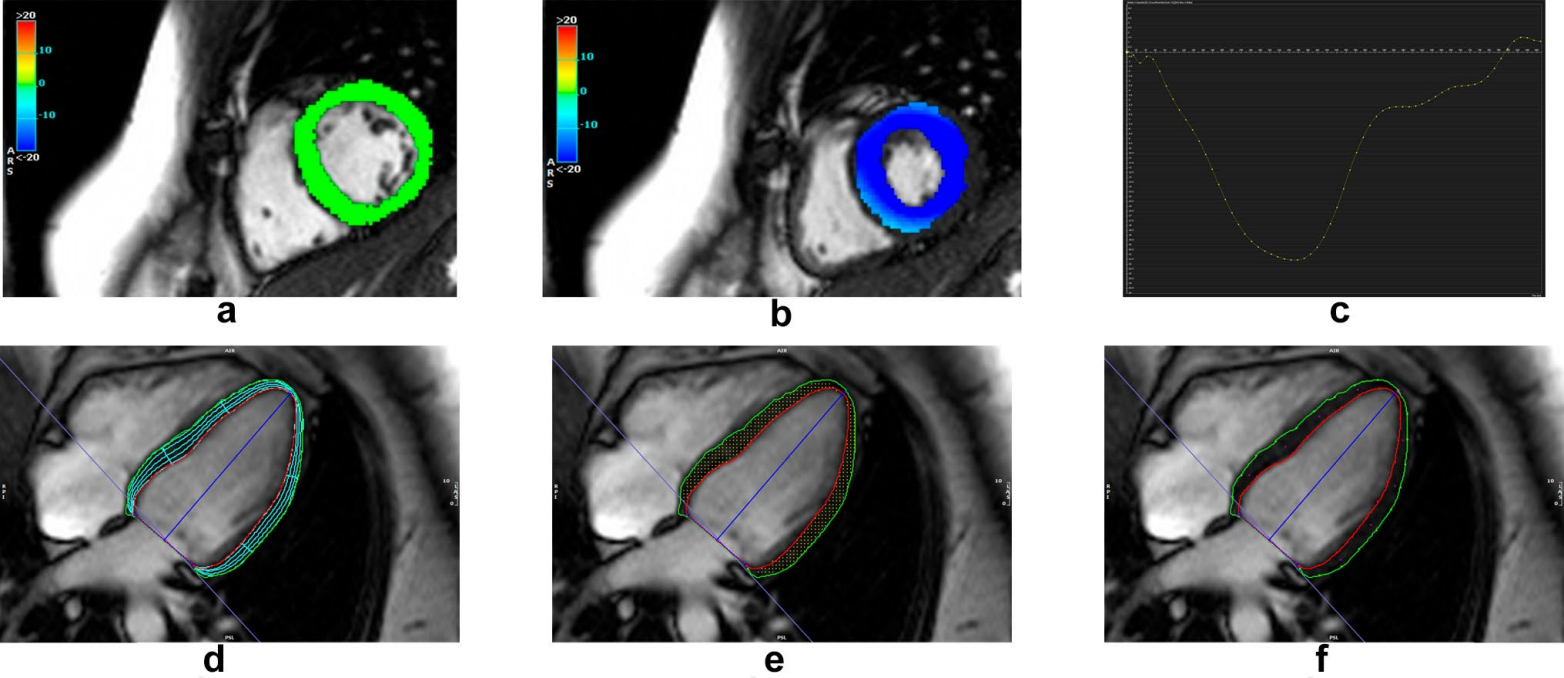
Feature Tracking Analysis by CVI^42^. Measurement of two-dimensional strain of the left ventricle. Circumferential strain assessment exemplary in a basal short-axis slice showing color-coded LV myocardium during diastole **a** and systole **b** and corresponding strain curve **c**. Quality assessment exemplary for accurate tracking and correct segmentation for longitudinal strain in 4-chamber view in long-axis using mesh function **d** and intramyocardial points **e, f**

Measurement of two-dimensional strain derived parameters was assessed performing a feature tracking (FT) analysis using CVI^42^ software (prototype version 5.3.0, Circle Cardiovascular Imaging Inc., Calgary, Canada) (Fig. 1). Circumferential strain and radial strain were obtained from short-axis stack analysis using full coverage while longitudinal strain was obtained from long-axis analysis using 2-chamber, 3-chamber and 4-chamber view. The comprehensive FT analysis has been published recently [41]. For the assessment of inter- and intraobserver agreement of global strain analysis, measurements were repeated in a randomly selected subsample (n = 10) by the same observer (intraobserver, C.B.) and by a different observer (interobserver, J.G.).

### Statistical analysis

Statistical analysis was performed using dedicated software (SPSS Statistics Version 27.0.0, IBM, Armonk, New York, USA). Continuous variables were expressed as mean ± standard deviation. The study population before and after 6-month dietary intervention was separately compared to healthy normal-weight controls using unpaired *t* tests. Paired *t* tests were conducted to test for differences between before and after 6-month dietary intervention separately for the low-carbohydrate and the low-fat diet group. Interaction between diet group assignment over 6 months (diet × time) was analyzed by employing a 2-way ANOVA for repeated measures. *p* < 0.05 was considered significant.

## Results

110 subjects with overweight to obesity without established cardiovascular disease completed the intervention phase [37]. In 95 of those, CMR images at baseline and after diet could be obtained. Five subjects were excluded due to significant artifacts related to impaired breath-holding at baseline or after diet, leaving a final intervention study cohort of 90 individuals. The control group consisted of 34 healthy volunteers (BMI < 25 kg/m^2^).

Characteristics of subjects with overweight to obesity without established cardiovascular disease irrespective of diet group and healthy normal-weight controls are given in Table 1. There was no significant age difference between study and control group.

**Table 1:** Baseline characteristics and cardiac parameters of people with overweight to obesity without established cardiovascular disease and healthy normal-weight controls

	People with Overweight to Obesity without established Cardiovascular Disease		Healthy Normal-Weight Controls	Student's *t*-test *p* value	
Variable	Baseline	After Diet		Baseline vs Controls	After Diet vs Controls
N (men/women)	90 (16/74)		34 (19/15)﻿		
Age, yrs	44.6 ± 9.3		﻿﻿40.8 ± 16.0	0.2317	﻿0.1685
Height, m	1.67 ± 0.08		﻿1.74 ± 0.08		
Body weight, kg	90.8 ± 15.9	84.9 ± 15.6	68.1 ± 8.5	< 0.0001	< 0.0001
BMI, kg/m^2^	32.6 ± 4	30.4 ± 4.2	22.5 ± 1.4	< 0.0001	< 0.0001
Systolic blood pressure, mm Hg	124 ± 12.3	117.8 ± 13.6	128.4 ± 15.1	0.1197	0.0005
Diastolic blood pressure, mm Hg	72.6 ± 7.5	68.8 ± 8.1	71.7 ± 12	0.6469	0.1333
Heart rate, beats/min	67.9 ± 9.1	63.4 ± 7.8	72.7 ± 11	0.0141	< 0.0001
LVEF,%	66 ± 6	65.4 ± 5.7	64.3 ± 4.1	0.0788	0.2570
LVEDV, ml	148.8 ± 28.2	147.1 ± 28.4	139.1 ± 27.4	0.0940	0.1719
Indexed LVEDV, ml/m	89 ± 13.5	87.8 ± 13.6	80 ± 14.3	0.0018	0.0069
LVSV, ml	97.9 ± 18.2	96 ± 19.5	89.3 ± 17.5	0.0222	0.0871
Indexed LVSV, ml/m	58.5 ± 8.8	57.3 ± 9.7	51.3 ± 9.2	0.0001	0.0030
LV mass, g	115.4 ± 27.1	103.7 ± 24	99 ± 14.2	< 0.0001	0.1961
Indexed LV mass, g/m	68.9 ± 13.7	61.8 ± 12	56.9 ± 7	< 0.0001	0.0085
LV mass/EDV, g/ml	0.77 ± 0.12	0.7 ± 0.1	0.73 ± 0.15	0.1036	0.3426
GCS, %	−20.1 ± 2	−19.5 ± 2	−17.8 ± 1.7	< 0.0001	< 0.0001
GRS, %	36.3 ± 6.1	34.2 ± 5.8	29.6 ± 4.5	< 0.0001	0.0001
GLS, %	−17.2 ± 1.8	−16.8 ± 1.9	−16.8 ± 1.8	0.2183	0.9290
LAEF, %	63.7 ± 5.6	63.8 ± 5.3	63.2 ± 7.2	0.7088	0.6812
LAEDV, ml	73.9 ± 17.5	68.2 ± 16.1	57.1 ± 11	< 0.0001	0.0006
Indexed LAEDV, ml/m	44.2 ± 9.6	40.7 ± 8.7	33 ± 6.4	< 0.0001	< 0.0001

Values are mean ± SD

*BMI* body mass index, *LVEF* left ventricular ejection fraction, *LVEDV* left ventricular end-diastolic volume, *LVSV* left ventricular stroke volume, *LV* left ventricular, *EDV* end-diastolic volume, *GCS* global circumferential strain, *GRS* global radial strain, *GLS* global longitudinal strain, *LAEF* left atrial ejection fraction, *LAEDV* left atrial end-diastolic volume

### LV strain

**Fig. 2:**
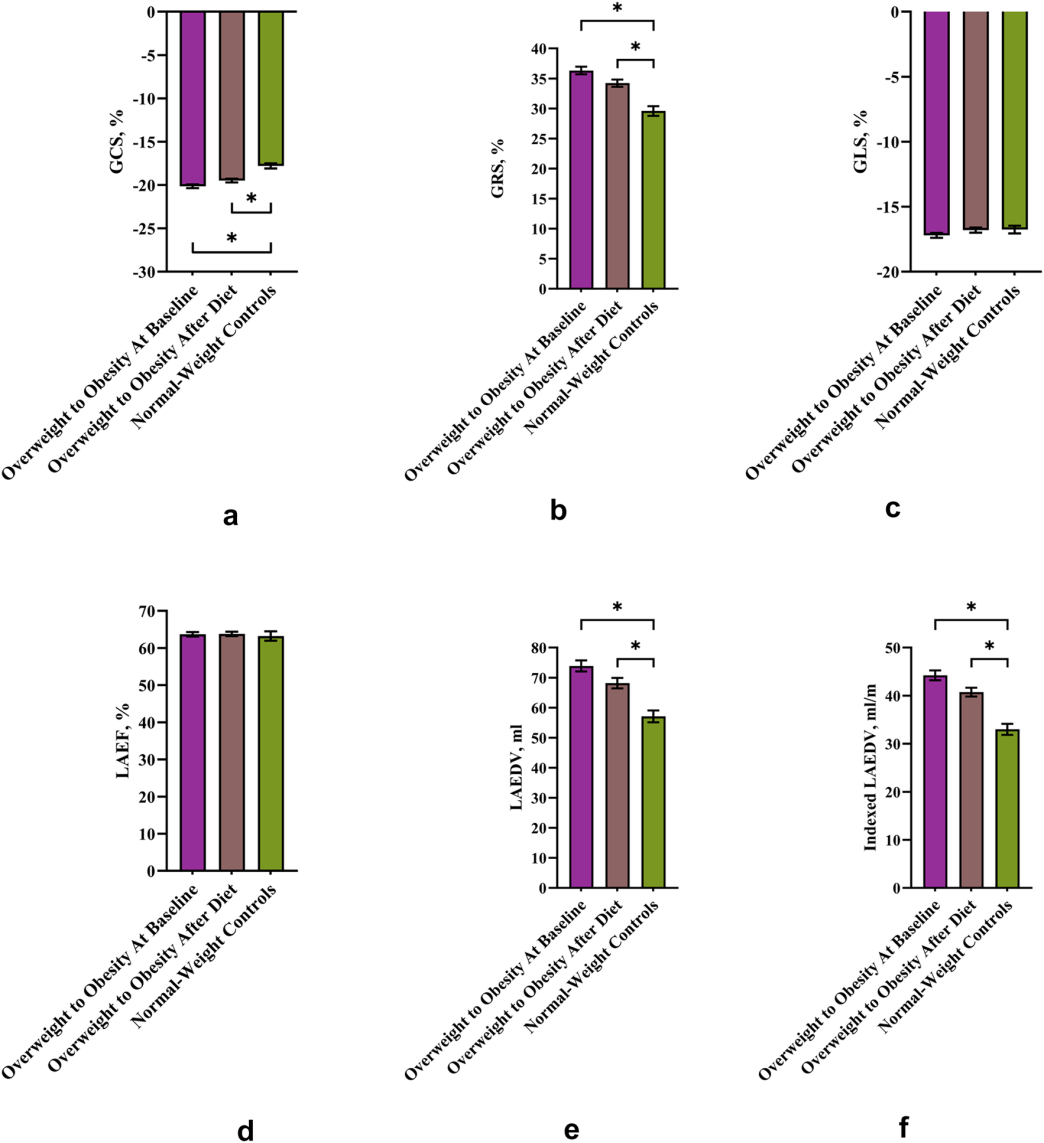
Global strain and LA size and function of study cohort with overweight to obesity versus normal-weight controls. Data are mean ± standard error of mean. Global circumferential strain (GCS) **a**, global radial strain (GRS) **b**, global longitudinal strain (GLS) **c**, left atrial ejection fraction (LAEF) **d**, left atrial end-diastolic volume (LAEDV) **e**, indexed LAEDV **f**. *p value < 0.05 as assessed by unpaired *t-*tests

GCS and GRS were significantly increased in the study cohort with overweight to obesity at baseline compared to the normal-weight control group (GCS −20.1 ± 2% vs −17.8 ± 1.7%, *p* < 0.0001; GRS 36.3 ± 6.1% vs 29.6 ± 4.5%, *p* < 0.0001) (Fig. 2a and b). After diet, GCS and GRS of the subjects with overweight to obesity still displayed significantly higher values compared to normal-weight controls (*p* < 0.0001 for GCS; *p* < 0.001 for GRS), but the difference to the normal-weight group was smaller compared to before diet. GLS did not differ between the normal-weight group and the study group, neither at baseline nor after diet (*p* > 0.05 for each comparison) (Fig. 2c).

**Fig. 3:**
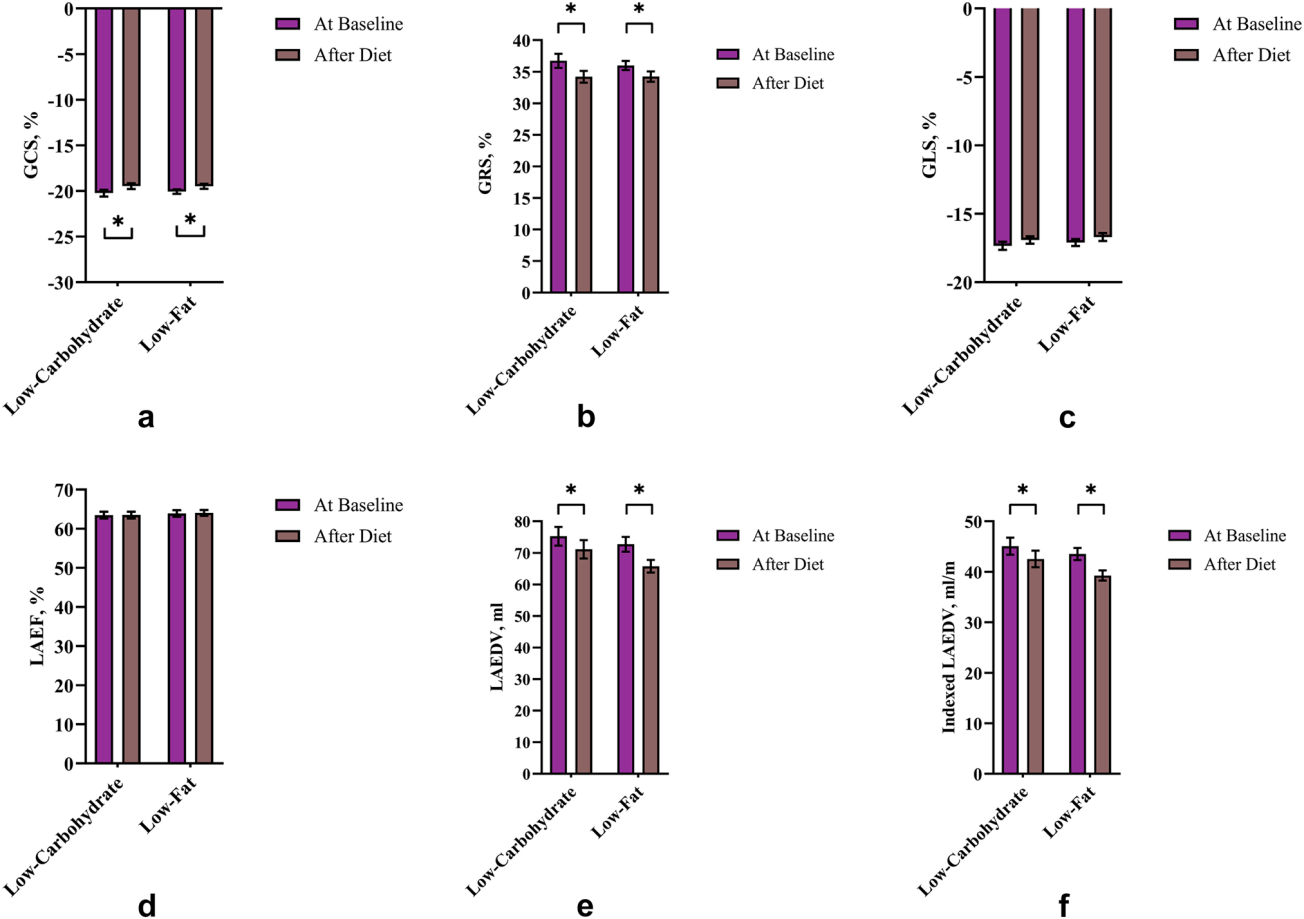
Diet effects on global strain and LA size and function of people with overweight to obesity without established cardiovascular disease. Data are mean ± standard error of mean. Global circumferential strain (GCS) **a**, global radial strain (GRS) **b**, global longitudinal strain (GLS) **c**, left atrial ejection fraction (LAEF) **d**, left atrial end-diastolic volume (LAEDV) **e**, indexed LAEDV **f**. *p value < 0.05 as assessed by paired *t-*tests

Within the study cohort on average both diet groups (low-fat and low-carbohydrate) showed a significant weight loss of approximately 6 kg with a 2 kg/m^2^ BMI reduction (Table 2). GCS decreased from approximately −20.1% at baseline to −19.5% after diet in both groups (*p* < 0.01 within each group; *p* interaction = 0.555) (Fig. 3a). At baseline, GRS values were on average 36.7% (low-carbohydrate group) and 36% (low-fat group) and were reduced after diet with average values of 34.2% (*p* < 0.01 within each group; *p* interaction = 0.395) (Fig. 3b). GLS did not differ between before diet (low-carbohydrate group −17.3 ± 1.9%; low-fat group −17.1 ± 1.7%) and after diet (low-carbohydrate group −16.9 ± 1.7%; low-fat group −16.7 ± 2%) irrespective of diet group (*p* > 0.05 within each group; *p* interaction = 0.962) (Fig. 3c).

**Table 2:** Diet effects on weight and cardiac parameters in people with overweight to obesity without established disease

Variable	Low-Carbohydrate		Student's *t*-test for paired samples* p* value	Low-Fat		Student's *t*-test for paired samples *p* value	2-Way ANOVA Diet × Time Interaction *p* value
	Baseline	After Diet		Baseline	After Diet		
N (men/women)	41 (6/35)			49 (10/39)			
Body weight, kg	90.5 ± 14.5	84.4 ± 14.1	< 0.0001	91.1 ± 17	85.3 ± 17	< 0.0001	0.787
BMI, kg/m^2^	32.5 ± 4.1	30.3 ± 4.4	< 0.0001	32.7 ± 3.9	30.5 ± 4	< 0.0001	0.998
Systolic blood pressure, mm Hg	120.9 ± 12.3	115.5 ± 12.5	0.0153	126.7 ± 11.9	119.8 ± 14.2	0.0001	0.549
Diastolic blood pressure, mm Hg	71.3 ± 7.5	67 ± 8	0.0018	73.7 ± 7.5	70.3 ± 7.9	0.0003	0.555
Heart rate, beats/min	70.3 ± 11.1	64.7 ± 7.8	0.0002	65.8 ± 6.4	62.3 ± 7.6	0.0004	0.191
LVEF, %	66.7 ± 5.9	65.6 ± 6	0.1733	65.5 ± 6	65.3 ± 5.5	0.7441	0.414
LVEDV, ml	149.4 ± 26	147.4 ± 29.9	0.2739	148.4 ± 30.2	146.8 ± 27.3	0.3737	0.893
Indexed LVEDV, ml/m	89.3 ± 13	88 ± 15	0.2074	88.7 ± 14.1	87.6 ± 12.5	0.3044	0.872
LVSV, ml	98.9 ± 15.3	96.5 ± 21.3	0.2401	97 ± 20.4	95.6 ± 18.1	0.4851	0.719
Indexed LVSV, ml/m	59.2 ± 7.7	57.6 ± 11.1	0.1899	57.9 ± 9.6	57 ± 8.5	0.4452	0.684
LV mass, g	115.5 ± 27.1	102.6 ± 23.4	< 0.0001	115.3 ± 27.3	104.6 ± 24.6	< 0.0001	0.252
Indexed LV mass, g/m	69 ± 14.5	61.1 ± 12.3	< 0.0001	68.8 ± 13.2	62.3 ± 11.8	< 0.0001	0.256
LV mass/EDV, g/ml	0.77 ± 0.14	0.7 ± 0.11	< 0.0001	0.78 ± 0.11	0.71 ± 0.09	< 0.0001	0.623
GCS, %	−20.2 ± 2.4	−19.5 ± 2.1	0.0031	−20.1 ± 1.6	−19.5 ± 2	0.0024	0.555
GRS, %	36.7 ± 7.2	34.2 ± 6	0.0012	36 ± 5.1	34.2 ± 5.7	0.0016	0.395
GLS, %	−17.3 ± 1.9	−16.9 ± 1.7	0.1651	−17.1 ± 1.7	−16.7 ± 2	0.0987	0.962
LAEF, %	63.5 ± 5.5	63.5 ± 5.4	0.8483	63.9 ± 5.7	64.1 ± 5.2	0.7993	0.754
LAEDV, ml	75.3 ± 18.9	71.1 ± 18.3	0.001	72.7 ± 16.3	65.8 ± 13.8	< 0.0001	0.166
Indexed LAEDV, ml/m	45.1 ± 10.9	42.5 ± 10.3	0.001	43.5 ± 8.4	39.3 ± 6.9	< 0.0001	0.171

Values are mean ± SD

*BMI* body mass index, *LVEF* left ventricular ejection fraction, *LVEDV* left ventricular end-diastolic volume, *LVSV* left ventricular stroke volume, *LV* left ventricular, *EDV* end-diastolic volume, *GCS* global circumferential strain, *GRS* global radial strain, *GLS* global longitudinal strain, *LAEF* left atrial ejection fraction, *LAEDV* left atrial end-diastolic volume

### LA size

Absolute and indexed LA end-diastolic volume were significantly larger in subjects with overweight to obesity at baseline compared to normal-weight subjects (absolute 73.9 ± 17.5 ml vs 57.1 ± 11 ml, *p* < 0.0001; indexed 44.2 ± 9.6 ml/m vs 33 ± 6.4 ml/m, *p* < 0.0001) (Fig. 2e and f). The difference in LA size between the study cohort after diet and normal-weight controls was still significant but smaller compared to the study cohort at baseline versus normal-weight controls (absolute 68.2 ± 16.1 ml vs 57.1 ± 11 ml, *p* < 0.001; indexed 40.7 ± 8.7 ml/m vs 33 ± 6.4 ml/m, *p* < 0.0001). LA ejection fraction was not different between the normal-weight and the study group, neither at baseline nor after diet (*p* > 0.05 for each comparison) (Fig. 2d).

Within the study group LA size was significantly reduced after diet in both diet groups compared to baseline: LA end-diastolic volume decreased about 4 ml in the low-carbohydrate group (*p* < 0.01) and about 7 ml in the low-fat group (*p* < 0.0001; *p* interaction = 0.166) (Fig. 3e). LA ejection fraction was approximately 64% and did not change significantly between before and after diet in neither group (*p* > 0.05 within each group; *p* interaction = 0.754) (Fig. 3d).

### LV mass and volumes

LV mass parameters were significantly higher in subjects with overweight to obesity at baseline compared to normal-weight controls (LV mass 115.4 ± 27.1 g vs 99 ± 14.2 g, *p* < 0.0001; indexed LV mass 68.9 ± 13.7 g/m vs 56.9 ± 7 g/m, *p* < 0.0001). After diet, absolute LV mass values of the study cohort were not different to those of normal-weight controls (103.7 ± 24 g vs 99 ± 14.2 g, *p* > 0.05). Indexed LV mass was still higher in the study cohort after diet compared to the normal-weight cohort but with a smaller difference to the normal-weight group compared to before diet (61.8 ± 12 g/m vs 56.9 ± 7 g/m, *p* < 0.01). No significant differences were found for LV ejection fraction and LV end-diastolic volume between normal-weight and diet group, neither at baseline nor after diet (*p* > 0.05 for each comparison). Indexed LV end-diastolic volume of subjects with overweight to obesity however displayed significantly higher values than normal-weight controls, both before and after diet (*p* < 0.01 for each comparison).

As shown previously by Haufe, Utz [37], a significant reduction in both diet groups after diet in comparison to baseline was seen in LV mass parameters: On average, absolute LV mass decreased after diet approximately 13 g in the low-carbohydrate group (115.5 ± 27.1 g vs 102.6 ± 23.4 g, *p* < 0.0001) and 11 g in the low-fat group (115.3 ± 27.3 g vs 104.6 ± 24.6 g, *p* < 0.0001; *p* interaction = 0.252). LV size remained unchanged in both diet groups (LV end-diastolic volume *p* > 0.05 within each group; *p* interaction = 0.893; LV ejection fraction *p* > 0.05 within each group; *p* interaction = 0.414). LV mass to volume ratio displayed significantly lower values after diet in comparison to baseline in both groups (*p* < 0.0001 within each group; *p* interaction = 0.623). Altogether no interaction effect between diet group assignment was found.

### Sex-related differences

We performed a subsample analysis for sex (Supplementary Table 2 for males, Supplementary Table 3 for females). For global strain values and LA size, the results between the normal-weight control group and people with overweight to obesity remained the same across sexes, meaning that GCS, GRS and LA size were significantly higher in the group with overweight to obesity irrespective of sex.

In the comparison between before and after diet, we found sex differences in GCS, GRS, GLS and LA size. The values were significantly reduced after diet in women (*p* < 0.05), while none of these were significantly changed in men (*p* > 0.05).

Within the study cohort we compared LV strain between men and women separately before and after diet (Supplementary Table 4). No significant sex differences were found.

### Intra- and inter-observer reproducibility for LV strain

Intra- and inter-observer agreements for LV strain parameters were high (C.B. and J.G.) (Table 3).

**Table 3:** Intra-observer and inter-observer reproducibility for LV strain parameters

Variable	Intra-observer	Inter-observer
	ICC (95% CI)	
GCS	0.972 (0.894 - 0.993)	0.973 (0.896 - 0.993)
Basal CS	0.922 (0.674 - 0.981)	0.977 (0.887 - 0.994)
Mid CS	0.926 (0.721 - 0.981)	0.958 (0.828 - 0.99)
Apical CS	0.855 (0.441 - 0.964)	0.797 (0.202 - 0.949)
GRS	0.973 (0.892 - 0.993)	0.971 (0.892 - 0.993)
Basal RS	0.912 (0.669 - 0.978)	0.972 (0.876 - 0.993)
Mid RS	0.923 (0.707 - 0.98)	0.954 (0.814 - 0.989)
Apical RS	0.826 (0.276 - 0.957)	0.828 (0.335 - 0.957)
GLS	0.880 (0.549 - 0.97)	0.902 (0.593 - 0.976)

*ICC* intraclass correlation coefficient, *CI* confidence interval, *GCS* global circumferential strain, *CS* circumferential strain, *GRS* global radial strain *RS* radial strain, *GLS* global longitudinal strain

## Discussion

We applied CMR FT based strain analysis to assess influences of obesity on myocardial deformation before and after dietary-induced weight loss. The important finding of our study is that GCS, GRS, LA volume and LV mass were all increased in people with overweight to obesity without established cardiovascular disease compared to healthy normal-weight controls. Weight loss through hypocaloric diet led to significant reductions in all these parameters irrespective of macronutrient composition. However, GCS, GRS, LV mass index, and LA size while being improved with modest weight loss remained elevated compared to healthy normal-weight controls.

### Obesity and myocardial deformation

CMR-FT studies in isolated obesity are rare. Deal, Rayner [39] observed GLS to be reduced in healthy obesity which was unaffected by dietary-induced weight loss. Likewise, another two studies found in their cross-sectional data a reduction of GLS and GCS in healthy people with obesity compared to healthy normal-weight controls [43, 44]. In contrast, our study findings suggest higher GCS and GRS and normal GLS in healthy obesity with dietary-induced changes towards GCS and GRS of healthy normal-weight controls. We cannot fully explain these discrepancies. Possibly, increased GCS and GRS in obesity without overt cardiovascular diseases might reflect a hypercontractile state as an adaptive cardiac response to increased circulating blood volume following obesity [3]. Though, dietary-induced reductions in myocardial deformation were not accompanied by reductions in LV stroke volume. Another possible explanation is that subclinical myocardial dysfunction, secondary to cardiovascular risk factors such as obesity, may primarily take place at the endocardial level resulting in a reduction in GLS with a compensatory GCS increase [45]. Following this, we found GCS to be increased in healthy obesity, though GLS was not decreased as expected. Zhang, Ma [46] showed in a cross-sectional cohort of normal-weight subjects that heart rate was positively correlated with GCS and not associated with GRS and GLS. As heart rate was significantly reduced after diet in our study cohort, a dietary-induced reduction in strain could be, at least for GCS, explained as a side effect of heart rate change. But this could not be applied to our control group who had a significantly higher heart rate and significantly lower values for GCS.

### Obesity and LA size

Assessment of LA size has been stated as an important variable for identifying diastolic dysfunction [47, 48], as LA dilatation is compensatory to higher LA pressures to maintain adequate LV filling [15]. LA enlargement was an independent predictor of cardiovascular events in several studies including stroke [49, 50], atrial fibrillation [51], acute myocardial infarction and cardiovascular death [52–54]. Previous studies showed that obesity is associated with atrial fibrillation [55, 56], LA enlargement [57, 58] and impaired LA strain [59]. Our study results confirmed an already increased LA volume in people with overweight to obesity without established cardiovascular disease while LA ejection fraction was not reduced, proposing an early stage of LA dysfunction in these subjects as impairment of LA ejection may be primarily in more advanced LA myopathy [60]. Increased LA size as an early sign of LA remodeling, as shown in our study, might be an important link between obesity without cardiovascular comorbidities and HF, as deteriorations in LA structure and function of asymptomatic individuals have been shown to precede development of HF [61]. Our results also imply beneficial effects of dietary-induced weight loss on LA remodeling in healthy obesity suggesting that LA enlargement in early stages is at least partially reversible [39].

### Sex-related differences

Based on subsample analysis for sex it could be identified that results differed between men and women within the study cohort between before and after diet. In men GCS, GRS and LA size were no longer significantly changed between before and after diet. We assume that those changes could also be related to the unequal sex distribution in our study cohort. Descriptively GCS and GRS and LA size were smaller after diet in men, but possibly did not reach statistical significance due to the small sample size. In women however additional to GCS, GRS and LA size, GLS was significantly reduced after diet in contrast to no significant GLS difference in the whole study cohort sample.

### Clinical implications

Our study findings have two clinical implications. Findings from our study suggest that subclinical abnormalities in myocardial deformation are already detectable in people with overweight to obesity without established cardiovascular disease. Dietary-induced weight loss leads to partial normalization of myocardial deformation. We propose that myocardial deformation through CMR-FT based strain analysis may have utility in predicting diastolic dysfunction and in targeting preventive measures in persons with overweight to obesity and may be further investigated in future studies.

### Study limitations

An important limitation of our study is that sex was not evenly distributed between groups with a significant higher female proportion in the study population and a slightly higher male proportion in the normal-weight control group. Some studies show no effect of sex on strain [62], yet other studies found partly sex differences for strain [63, 64]. Physical activity was not directly monitored, however, our study dieticians reminded participants to kee*p* their physical activity constant. We cannot exclude that changing dietary habits might have influenced other health behaviors such as physical activity. Finally, our data assessment was retrospective.

## Conclusions

In summary, overweight and obesity in otherwise healthy subjects are significantly associated with increased GCS, GRS and LA size. Dietary-induced weight loss significantly decreases GCS, GRS and LA size irrespective of macronutrient composition leading to a partial normalization of these parameters.

### Supplementary Information

Below is the link to the electronic supplementary material.

Supplementary file1 (DOCX 28 kb)

## Abbreviations

BMI: Body mass index
CMR: Cardiovascular magnetic resonance
FT: Feature tracking
GCS: Global circumferential strain
GLS: Global longitudinal strain
GRS: Global radial strain
HF: Heart failure
HFpEF: Heart failure with preserved ejection fraction
LA: Left atrial
LV: Left ventricular
