# Supplementary material for: Adiposity influences on myocardial deformation: a cardiovascular magnetic resonance feature tracking study in people with overweight to obesity without established cardiovascular disease
Source: Int J Cardiovasc Imaging. 2024 Feb 2;40(3):643–54. doi: 10.1007/s10554-023-03034-2 (PMC10951011; doi:10.1007/s10554-023-03034-2)
Supplement: Supplementary file 1 — Supplementary file1 (DOCX 28 kb) [file 10554_2023_3034_MOESM1_ESM.docx]

Supplementary material for “Adiposity influences on myocardial deformation: a cardiovascular magnetic resonance feature tracking study in people with overweight to obesity without established cardiovascular disease“

| Supplementary Table 1 Strain Rates (SR) in People with Overweight to Obesity without Established Cardiovascular Before and After Diet | | | |
| --- | --- | --- | --- |
| Variable | Baseline | After Diet | Student’s t-test  p value  Baseline vs After Diet |
| N | 90 | |  |
| Circumferential Systolic SR, s^-1^ | -1.44 ± 1.32 | -1.22 ± 0.39 | 0.124 |
| Radial Systolic SR, s^-1^ | 2.47 ± 1.35 | 2.31 ± 0.50 | 0.239 |
| Longitudinal Systolic SR, s^-1^ | -1.05 ± 0.54 | -1.35 ±3.24 | 0.398 |
| Circumferential Diastolic SR, s^-1^ | 1.36 ± 0.35 | 1.26 ± 0.24 | 0.001 |
| Radial Diastolic SR, s^-1^ | -2.57 ± 0.72 | -2.29 ± 0.59 | <0.0001 |
| Longitudinal Diastolic SR, s^-1^ | 1.18 ± 0.24 | 1.11 ± 0.32 | 0.058 |
| Values are mean ± SD. SR = strain rate | | | |

| **Supplementary Table 2** Baseline Characteristics and Cardiac Parameters of Men with Overweight to Obesity without Established Cardiovascular and Healthy Normal-Weight | | | | | | | | |
| --- | --- | --- | --- | --- | --- | --- | --- | --- |
|  | Men with Overweight to Obesity | |  | Healthy Normal-Weight Men |  | Student’s t-test / Mann-Whitney-U-Test  p value | | |
| Variable | Baseline | After Diet |  |  |  | Baseline vs After Diet | Baseline vs Controls | After Diet vs Controls |
| N | 16 | |  | 19 |  |  |  |  |
| Age, yrs | 46.7 ± 9.3 | |  | 34.9 ± 15.5 |  |  |  |  |
| Height, m | 1.77 ± 0.05 | |  | 1.77 ± 0.09 |  |  |  |  |
| Body weight, kg | 106.6 ± 18.7 | 99.5 ± 20.4 |  | 72.7 ± 7.9 |  | 0.003 | <0.0001 | <0.0001 |
| BMI, kg/m^2^ | 33.8 ± 5.3 | 31.3 ± 6.0 |  | 23.0 ± 1.3 |  | 0.002 | <0.0001 | <0.0001 |
| Systolic blood pressure, mm Hg | 127.4 ± 14.9 | 122.2 ± 15.9 |  | 126.4 ± 10.8 |  | 0.146 | 0.812 | 0.383 |
| Diastolic blood pressure, mm Hg | 74.6 ± 9.5 | 72.5 ± 8.2 |  | 71.2 ± 14 |  | 0.289 | 0.321^m^ | 0.894^m^ |
| Heart rate, beats/min | 64.2 ± 6.9 | 61.5 ± 7.3 |  | 71.9 ± 12.3 |  | 0.148 | 0.051^m^ | 0.002^m^ |
| LVEF, % | 66.3 ± 5.6 | 65.7 ± 5.7 |  | 64.7 ± 4.6 |  | 0.632 | 0.383 | 0.593 |
| LVEDV, ml | 181.7 ± 34.0 | 181.7 ± 31.3 |  | 155.3 ± 20.1 |  | 0.998 | 0.012 | 0.008 |
| Indexed LVEDV, ml/m | 102.2 ± 17.1 | 101.9 ± 16.8 |  | 87.6 ± 11.0 |  | 0.912 | 0.007 | 0.008 |
| LVSV, ml | 119.8 ± 20.3 | 118.9 ± 20.6 |  | 100.1 ± 11.9 |  | 0.860 | 0.001 | 0.002 |
| Indexed LVSV, ml/m | 67.4 ± 10.3 | 66.7 ± 11.6 |  | 56.5 ± 6.6 |  | 0.809 | 0.001 | 0.003 |
| LV mass, g | 155.2 ± 30.1 | 140.4 ± 23.3 |  | 104.9 ± 13.3 |  | <0.0001 | <0.0001 | <0.0001 |
| Indexed LV mass, g/m | 87.5 ± 16.7 | 78.8 ± 12.8 |  | 59.1 ± 6.4 |  | <0.0001 | <0.0001 | <0.0001 |
| LV mass/EDV, g/ml | 0.85 ± 0.16 | 0.78 ± 0.12 |  | 0.68 ± 0.09 |  | 0.006 | <0.0001 | 0.017 |
| GCS, % | -19.6 ± 1.7 | -19.5 ± 1.6 |  | -17.5 ± 1.7 |  | 0.635 | 0.001 | 0.001 |
| GRS, % | 34.8 ± 5.7 | 33.8 ± 4.8 |  | 28.7 ± 4.1 |  | 0.340 | 0.001 | 0.002 |
| GLS, % | -16.4 ± 1.5 | -16.4 ± 1.4 |  | -16.4 ± 2.0 |  | 0.906 | 0.914 | 0.988 |
| LAEF, % | 63.3 ± 4.7 | 63.8 ± 5.3 |  | 62.6 ± 7.2 |  | 0.112 | 0.721 | 0.999^m^ |
| LAEDV, ml | 83.0 ± 18.2 | 79.5 ± 15.1 |  | 56.9 ± 10.9 |  | 0.097 | <0.0001 | <0.0001 |
| Indexed LAEDV, ml/m | 46.7 ± 9.7 | 44.5 ± 8.3 |  | 32.2 ± 6.2 |  | 0.079 | <0.0001 | <0.0001 |
| values are mean ± SD. ^m^ means use of Mann-Whitney-U test if the variable was not normally distributed.  BMI = body mass index; LVEF = left ventricular ejection fraction; LVEDV = left ventricular end-diastolic volume; LVSV = left ventricular stroke volume; LV = left ventricular; EDV = end-diastolic volume; GCS = global circumferential strain; GRS = global radial strain; GLS = global longitudinal strain; LAEF = left atrial ejection fraction; LAEDV = left atrial end-diastolic volume. | | | | | | | | |

| **Supplementary Table 3** Baseline Characteristics and Cardiac Parameters of Women with Overweight to Obesity without Established Cardiovascular and Healthy Normal-Weight | | | | | | | | |
| --- | --- | --- | --- | --- | --- | --- | --- | --- |
|  | Women with Overweight to Obesity | |  | Healthy Normal-Weight Women |  | Student’s t-test  p value | | |
| Variable | Baseline | After Diet |  |  |  | Baseline vs After Diet | Baseline vs Controls | After Diet vs Controls |
| N | 74 | |  | 15 |  |  |  |  |
| Age, yrs | 43.8 ± 9.3 | |  | 48.3 ± 13.6 |  |  |  |  |
| Height, m | 1.64 ± 0.07 | |  | 1.68 ± 0.04 |  |  |  |  |
| Body weight, kg | 87.4 ± 13.0 | 81.7 ± 12.5 |  | 62.3 ± 5.1 |  | <0.0001 | <0.0001^m^ | <0.0001^m^ |
| BMI, kg/m^2^ | 32.3 ± 3.6 | 30.2 ± 3.7 |  | 21.8 ± 1.4 |  | <0.0001 | <0.0001^m^ | <0.0001^m^ |
| Systolic blood pressure, mm Hg | 123.3 ± 11.7 | 116.9 ± 12.9 |  | 131.0 ± 19.6 |  | <0.0001 | 0.217 ^m^ | 0.004^m^ |
| Diastolic blood pressure, mm Hg | 72.2 ± 7.1 | 68.0 ± 7.9 |  | 73.8 ± 8.8 |  | <0.0001 | 0.565^m^ | 0.028^m^ |
| Heart rate, beats/min | 68.7 ± 9.3 | 63.8 ± 7.8 |  | 73.7 ± 9.5 |  | <0.0001 | 0.040^m^ | <0.0001 |
| LVEF, % | 66.0 ± 6.1 | 65.3 ± 5.7 |  | 63.8 ± 3.5 |  | 0.283 | 0.074 | 0.392 ^m^ |
| LVEDV, ml | 141.7 ± 21.1 | 139.6 ± 21.5 |  | 118.4 ± 20.8 |  | 0.061 | <0.0001 | 0.001 |
| Indexed LVEDV, ml/m | 86.1 ± 10.8 | 84.70 ± 10.7 |  | 70.2 ± 12.1 |  | 0.047 | <0.0001 | <0.0001 |
| LVSV, ml | 93.1 ± 13.8 | 91.1 ± 15.3 |  | 75.4 ± 13.1 |  | 0.134 | <0.0001 | 0.001 |
| Indexed LVSV, ml/m | 56.6 ± 7.1 | 55.3 ± 8.0 |  | 44.7 ± 7.7 |  | 0.113 | <0.0001 | <0.0001 |
| LV mass, g | 107.0 ± 17.3 | 95.9 ± 15.4 |  | 91.4 ± 11.8 |  | <0.0001 | 0.002^m^ | 0.560 ^m^ |
| Indexed LV mass, g/m | 65.0 ± 9.1 | 58.2 ± 8.1 |  | 54.2 ± 7.1 |  | <0.0001 | <0.0001^m^ | 0.255 ^m^ |
| LV mass/EDV, g/ml | 0.76 ± 0.10 | 0.69 ± 0.09 |  | 0.79 ± 0.18 |  | <0.0001 | 0.441 | 0.045 |
| GCS, % | -20.3 ± 2.0 | -19.4 ± 2.1 |  | -18.1 ± 1.7 |  | <0.0001 | 0.001 | 0.033 |
| GRS, % | 36.7 ± 6.2 | 34.3 ± 6.0 |  | 30.7 ± 4.9 |  | <0.0001 | 0.001 | 0.039 |
| GLS, % | -17.4 ± 1.7 | -16.8 ± 1.9 |  | -17.3 ± 1.9 |  | 0.019 | 0.806 | 0.504 |
| LAEF, % | 63.8 ± 5.8 | 64.3 ± 5.3 |  | 64.1 ± 7.4 |  | 0.357 | 0.857 | 0.915 |
| LAEDV, ml | 71.9 ± 16.8 | 65.8 ± 15.4 |  | 57.4 ± 11.5 |  | <0.0001 | 0.001^m^ | 0.032^m^ |
| Indexed LAEDV, ml/m | 43.7 ± 9.6 | 39.9 ± 8.6 |  | 34.0 ± 6.8 |  | <0.0001 | <0.0001^m^ | 0.010^m^ |
| Values are mean ± SD. ^m^ means use of Mann-Whitney-U test if the variable was not normally distributed.  BMI = body mass index; LVEF = left ventricular ejection fraction; LVEDV = left ventricular end-diastolic volume; LVSV = left ventricular stroke volume; LV = left ventricular; EDV = end-diastolic volume; GCS = global circumferential strain; GRS = global radial strain; GLS = global longitudinal strain; LAEF = left atrial ejection fraction; LAEDV = left atrial end-diastolic volume. | | | | | | | | |

| **Supplementary Table 4** LV Strain for Men and Women with with Overweight to Obesity without Established Cardiovascular Disease | | | | | | |
| --- | --- | --- | --- | --- | --- | --- |
|  | Before Diet | | Student’s t-test  p value | After Diet | | Student’s  t-test  p value |
| Variable | Men | Women |  | Men | Women |  |
| GCS, % | -19.6 ± 1.7 | -20.3 ± 2.0 | 0.250 | -19.5 ± 1.6 | -19.4 ± 2.1 | 0.959 |
| GRS, % | 34.8 ± 5.7 | 36.7 ± 6.2 | 0.274 | 33.8 ± 4.8 | 34.3 ± 6.0 | 0.736 |
| GLS, % | -16.4 ± 1.5 | -17.4 ± 1.7 | 0.061 | -16.4 ± 1.4 | -16.8 ± 1.9 | 0.332 |
| Values are mean ± SD.  GCS = global circumferential strain; GRS = global radial strain; GLS = global longitudinal strain. | | | | | | |
